# The Missing Men: HIV Treatment Scale-Up and Life Expectancy in Sub-Saharan Africa

**DOI:** 10.1371/journal.pmed.1001906

**Published:** 2015-11-24

**Authors:** Alexander C. Tsai, Mark J. Siedner

**Affiliations:** 1 Department of Psychiatry, Massachusetts General Hospital, Boston, Massachusetts, United States of America; 2 Mbarara University of Science and Technology, Mbarara, Uganda; 3 Harvard Medical School, Boston, Massachusetts, United States of America; 4 Division of Infectious Diseases, Department of Medicine, Massachusetts General Hospital, Boston, Massachusetts, United States of America

## Abstract

In a Perspective accompanying Bor and colleagues, Alexander Tsai and Mark Siedner discuss the gender gap in ART uptake and HIV mortality in Africa.

Delivery of effective HIV antiretroviral therapy (ART) to the more than 6 million persons with HIV in South Africa is well underway, with early data on the impact of this massive public health effort demonstrating a reversal of the previous decade’s precipitous decline in population life expectancy [1]. Although South Africa’s age and sex disparities in HIV acquisition have traditionally been described as disadvantaging young women [2], accumulating evidence now suggests a reverse disparity: although HIV care is available to both men and women and is nominally free of charge, women are more likely to be tested for HIV, engage in pre-treatment care, initiate treatment earlier, stay on treatment, and survive [3–6]. To adopt the classic Eisenberg and Power [7] analogy of health care as current flowing through an electric circuit, the voltage drops along the entire circuit of HIV care, from HIV infection to AIDS-free survival, are larger for men compared with women (**Fig 1**). There are simply too many missing men.

**Fig 1:**
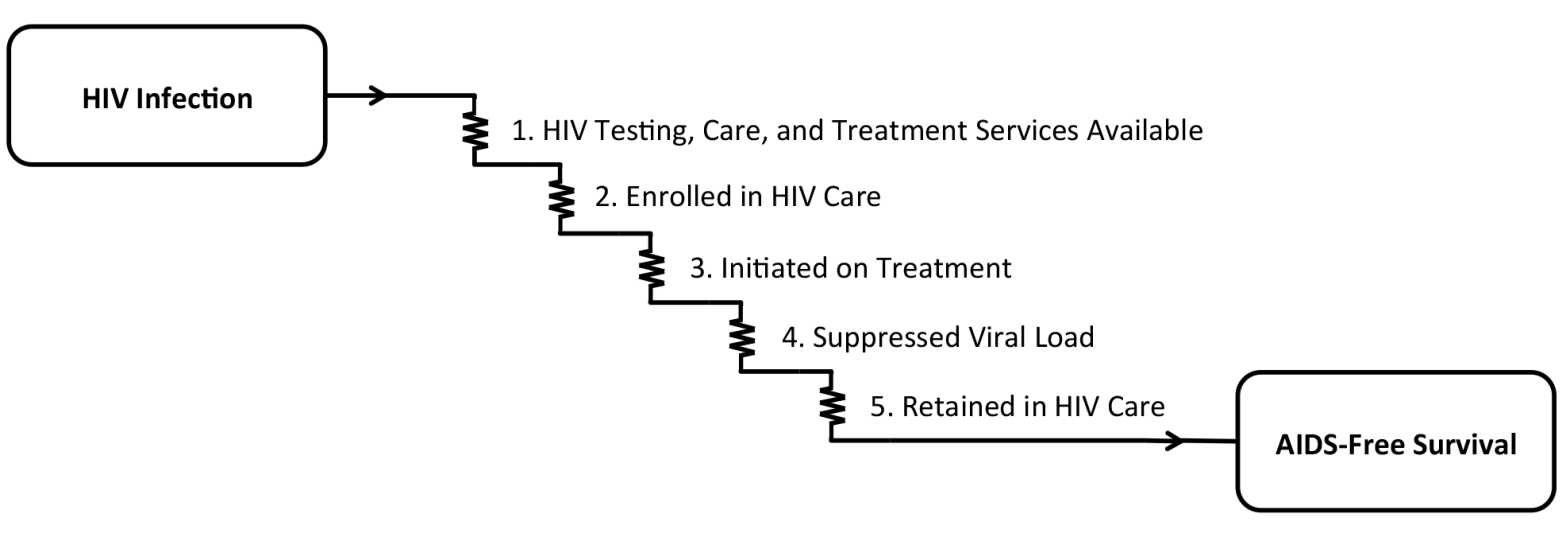
The cascade of “voltage drops” from HIV infection to AIDS-free survival. In order for the goal of AIDS-free survival to be achieved, (1) HIV testing, care, and treatment services must be available, and persons with HIV must (2) be enrolled in care, (3) initiate antiretroviral therapy, (4) achieve suppression of HIV-1 RNA viral load, and (5) be retained in care.

In recent years, studies from South Africa [8], as well as Rwanda [9] and Uganda [10], have begun to demonstrate the cumulative impact of these voltage drops, which, in total, result in an approximately 10-year life expectancy gap between men and women initiating ART at 20 years of age (**Fig 2**). However, the findings of these studies should be interpreted in light of important limitations. First, they were based solely on data obtained from persons enrolled in HIV treatment programs. Poverty, food insecurity, HIV stigma, and geographic barriers still exert outsize influences on HIV testing, treatment, and retention in these settings [11–14], so it is unlikely that these enrollees are representative of the entire population of persons with HIV. Second, mortality had to be estimated among those lost to follow up [9,10]. Because persons in HIV treatment programs are much more likely to be lost to care than confirmed as dead [15,16], and because the vast majority of HIV-related mortality events go unreported [17], the mortality estimates in these studies are likely to be biased. Third, and perhaps most notably, none of these studies directly observed non-HIV mortality. Thus, while they were able to document trends in mortality among persons with HIV, they were unable to assess the extent to which these changes were related to HIV care or to unrelated secular trends in health and health behavior.

**Fig 2:**
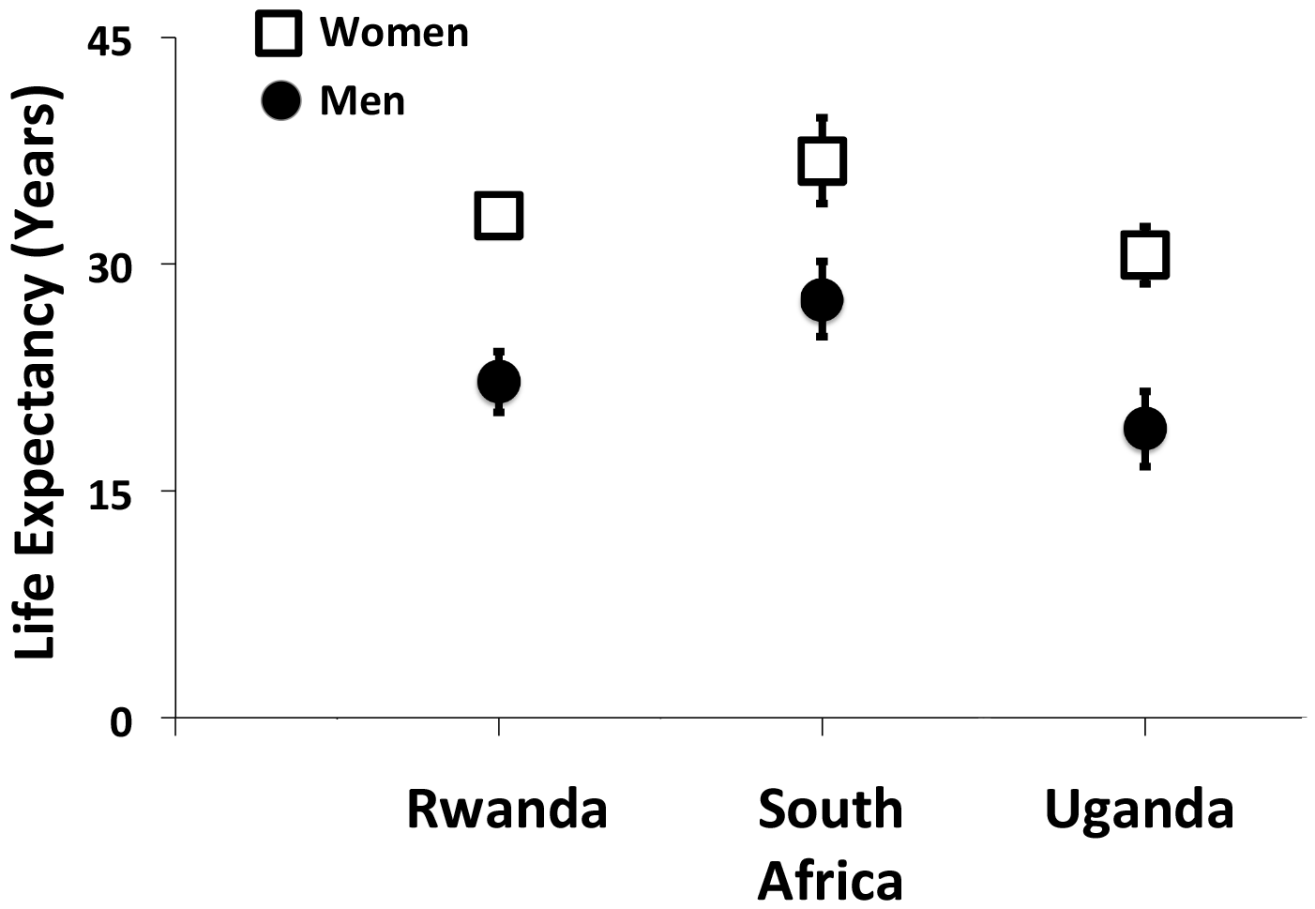
Gender gaps in life expectancy among men and women with HIV initiating antiretroviral therapy at 20 years of age. This figure summarizes the findings of studies from Rwanda [9], South Africa [8], and Uganda [10]. Estimates and associated 95% confidence intervals are shown as the number of additional years of life expected for men and women with HIV initiating antiretroviral therapy at 20 years of age.

In this context, the research article by Jacob Bor and colleagues [18] that appears this week in *PLOS Medicine* provides new evidence of a widening gender gap in life expectancy, using data obtained from a general population sample in rural South Africa from 2001–2011, covering a period of coincident ART scale-up. By surveilling all persons in the region—whether HIV-negative, HIV-positive in care, or HIV-positive but not in care—and by using verbal autopsies to categorize mortality events that were recorded by the surveillance teams, this study addresses some of the limitations of previous work. Subject to the assumption that mortality events were comprehensively observed and accurately categorized—which would be generically limiting for any study conducted in a country with less than complete registration of vital events [19]—Bor and colleagues [18] were not obligated to account for non-uptake of HIV testing, nor were they required to estimate mortality among those lost to care or to estimate non-HIV mortality. Their findings are summarized by the stark observation that life expectancy gains among women far outstripped the life expectancy gains among men, and that these gains were independent of both age and first recorded CD4+ T-lymphocyte cell count. Perhaps more telling than the near-doubling of the gender gap in life expectancy during the observation period are the relative benefits women received throughout the entire HIV care circuit: HIV-related mortality rates were approximately 2-fold higher among men compared with women, whether prior to ART or during both early and long-term ART.

What could explain these findings? Certainly women’s differential access to HIV care during pregnancy might be partially responsible. South Africa’s successful program for preventing mother-to-child transmission largely requires HIV testing for all pregnant women and encourages ART initiation among those found to be HIV positive [20]. This institutional link to program entry could partially explain why more than half of HIV-related mortality events among men in 2007–2011 occurred during the pre-treatment period, compared with only one-third of HIV-related mortality events among women. However, because the lower HIV-related mortality rates among women persisted even after accounting for age, CD4 count, and ART initiation, clearly more data are needed to explain the widening gender gap in life expectancy. Other major contributions likely result from historically ingrained social forces (such as increased migratory needs resulting from apartheid) and differential patterns of health behavior [21–23]. Even in the absence of these crippling disadvantages, a gender gap in life expectancy may yet remain [24], but the data presented by Bor and colleagues [18] signal the urgent need to better understand this large and widening disparity in South Africa and elsewhere in sub-Saharan Africa.

What can be done to address this problem? Different types of interventions should be considered. Minimally, policies could be revised to “nudge” men into HIV care; for example, opt-out HIV testing among military service members could be mandated as part of annual examinations or after deployments, peacekeeping missions, or foreign trainings [25]. Home-based HIV counseling and testing can potentially provide a greater degree of privacy for men concerned about discrimination, or by providing convenience for men whose willingness to undergo testing is constrained by work obligations [26]. Similarly, workplace-based treatment programs [27] or alternative patient-centered care models [28] may help to retain men in care once treatment has been initiated. And finally, social marketing to emphasize collateral impacts—such as economic benefits for individuals and their households [29] or reduced risks of secondary transmission to domestic partners [30] and/or unborn children [31]—may provide additional impetus for testing and treatment. Of note, while these “gender sensitive” intervention strategies attempt to minimize the ways in which socially constructed gender roles in South Africa constrain men’s health behavior, they still leave intact a system of gender inequality that confers distinct health disadvantages for women while simultaneously marshaling other threats to the health of men. Truly “gender transformative” intervention strategies will need to understand men’s health behavior as being intimately tied to the same prevailing gender roles and norms of masculinity that produce violence against women, constraints on capital ownership, alcohol and substance abuse, and sexual risk taking [23]. Given the complexity of the problem, multipronged approaches will likely be needed. Certainly, the AIDS-free generation will remain a far-off mirage until men also receive the health benefits made possible through the mass provision of HIV treatment, which somehow remains out of reach for too many of them.

## Abbreviations

ART: antiretroviral therapy
